# *Aspergillus fumigatus* Infection-Induced Neutrophil Recruitment and Location in the Conducting Airway of Immunocompetent, Neutropenic, and Immunosuppressed Mice

**DOI:** 10.1155/2018/5379085

**Published:** 2018-01-18

**Authors:** Marina A. Shevchenko, Andrey O. Bogorodskiy, Natalia I. Troyanova, Ekaterina A. Servuli, Elena L. Bolkhovitina, Georg Büldt, Christoph Fahlke, Valentin I. Gordeliy, Thomas Gensch, Valentin I. Borshchevskiy, Alexander M. Sapozhnikov

**Affiliations:** ^1^Laboratory of Cell Interactions, Department of Immunology, Shemyakin and Ovchinnikov Institute of Bioorganic Chemistry, Russian Academy of Sciences, Miklukho-Maklaya St. 16/10, Moscow 117997, Russia; ^2^Laboratory for Advanced Studies of Membrane Proteins, Moscow Institute of Physics and Technology, 9 Institutskiy Per., Dolgoprudny 141701, Russia; ^3^Institute of Complex Systems 4 (ICS-4: Cellular Biophysics), Forschungszentrum Jülich, 52425 Jülich, Germany; ^4^Institute of Complex Systems 6 (ICS-6: Structural Biochemistry), Forschungszentrum Jülich, 52425 Jülich, Germany; ^5^Université Grenoble Alpes, CEA, CNRS, IBS, 38000 Grenoble, France

## Abstract

Susceptibility to fungal infection is commonly associated with impaired neutrophil responses. To study the mechanisms underlying this association, we investigated neutrophil recruitment to the conducting airway wall after *Aspergillus fumigatus* conidium inhalation in mouse models of drug-induced immunosuppression and antibody-mediated neutrophil depletion (neutropenia) by performing three-dimensional confocal laser-scanning microscopy of whole-mount primary bronchus specimens. Actin staining enabled visualization of the epithelial and smooth muscle layers that mark the airway wall. Gr-1^+^ or Ly6G^+^ neutrophils located between the epithelium and smooth muscles were considered airway wall neutrophils. The number of airway wall neutrophils for immunocompetent, immunosuppressed, and neutropenic mice before and 6 h after *A. fumigatus* infection were analyzed and compared. Our results show that the number of conducting airway wall neutrophils in immunocompetent mice significantly increased upon inflammation, while a dramatic reduction in this number was observed following immunosuppression and neutropenia. Interestingly, a slight increase in the infiltration of neutrophils into the airway wall was detected as a result of infection, even in immunosuppressed and neutropenic mice. Taken together, these data indicate that neutrophils are present in intact conducting airway walls and the number elevates upon *A. fumigatus* infection. Conducting airway wall neutrophils are affected by both neutropenia and immunosuppression.

## 1. Introduction

*Aspergillus fumigatus* is an airborne pathogen that causes a life-threatening condition for patients receiving immunosuppressive therapy or people with congenital neutrophil dysfunction and can also be harmful to individuals with allergies of different etiology [1, 2]. Multiple studies have confirmed that an insufficient neutrophil response is the primary factor underlying this increased susceptibility to fungal infection [3–5]. In fact, extensive neutrophil recruitment to *A. fumigatus*-infected mouse lungs was observed as early as 6 h postinfection using mouse models and multiple detection techniques, including bronchoalveolar lavage cell count, flow cytometry of homogenized lung tissue suspensions, and histological examination of both fixed and live lung slices [6–9]. The main source of these neutrophils is the bone marrow, and upon infection, they are mobilized to the blood vessels [10], allowing them to migrate to infected lung tissue [11–13]. These circulating and vascular marginated pools of neutrophils provide a reasonably fast immune response to pathogen invasion [14]. Neutrophils have also been identified in the interstitial compartments of uninjured mouse lungs and are the primary type of leukocyte recruited to the interstitial compartment during inflammation [15, 16]. Neutrophil potential to *A. fumigatus* conidium internalization was demonstrated both *in vitro* and in bronchoalveolar lavages and in alveolar compartment of infected mouse lungs [6, 7, 9]. Despite the strong evidence of neutrophil–conidium interaction, the anatomical location of neutrophils recruited to the lungs during *A. fumigatus* infection has not been fully characterized. Researchers mostly reported increase in neutrophil numbers in bronchoalveolar lavages or whole lung tissue homogenates [6–8], while data about distribution of neutrophils in the different lung microcompartments, especially at the sites of potential interaction to *A. fumigatus* conidia, are the most useful for understanding the consequence of events during pathogen recognition and clearance. Upon inhalation, conidia are transferred by airflow to the airway lumen. To sense and interact with them, neutrophil should reside in the airway wall—the microcompartment that starts from the luminal side of the epithelial barrier and ends at the lateral side of the smooth muscle layer [17]. Although neutrophil infiltration to the airway wall was observed in different airway inflammatory responses [18, 19], such recruitment of neutrophils has not been investigated during *A. fumigatus* infection.

In the present study, we used whole-mount immunofluorescent staining and confocal laser scanning microscopy (LSM) to acquire three-dimensional images of mucosa-associated epithelium, smooth muscles, and neutrophils in *A. fumigatus*-infected and noninfected conducting airways. The number of neutrophils located between the epithelial and smooth muscle layers was estimated for intact mice, for mice that received a combination of an antineoplastic drug and a corticosteroid or mice that were injected with anti-Gr-1 or anti-Ly6G neutrophil-depleting antibodies. Neutrophil recruitment was also analyzed in *A. fumigatus*-infected mice with induced immunosuppression and neutropenia. Since the germination of conidia is associated with unmasking of pattern-recognition receptors and subsequent activation of macrophage-mediated response [20, 21], to exclude excessive immune system activation, we used paraformaldehyde-fixed conidia that mimicked the dormant state of spores. To our knowledge, this is the first study to investigate the localization and to quantify conducting airway wall neutrophils in different immunosuppressive states and during *A. fumigatus* infection.

## 2. Materials and Methods

### 2.1. Animals and Ethics Statement

Female BALB/c mice (10–14 weeks old) were obtained from the Pushchino Animal Breeding Centre (Russia) for this study. All animal experiments were performed in concordance with the Guide for the Care and Use of Laboratory Animals under a protocol approved by the Institutional Animal Care and Use Committee at the Shemyakin–Ovchinnikov Institute of Bioorganic Chemistry, Russian Academy of Sciences (protocol numbers 205/2016 and 226/2017). Animals were given standard food and tap water ad libitum and housed under regular 12 h dark : light cycles at 22°C.

### 2.2. *Aspergillus fumigatus* Strain, Media, and Growth Conditions

The *A. fumigatus* strain AfS150 [22], generated from ATCC 46645 and expressing the dTomato fluorescent protein, was used in this study. The fungus was grown at 37°C on *Aspergillus* minimal medium supplemented with 1% D-glucose as the carbon source. A fungal suspension was transferred to AMM agar plates and incubated for 3 days at 37°C. Conidia were harvested in 0.01% Tween 20–Dulbecco's Phosphate-Buffered Saline (DPBS) (PanEco, Russia) solution.

Conidia were then fixed overnight with 3% paraformaldehyde (Sigma-Aldrich, USA), washed twice with DPBS, filtered through Steriflip Filter Units (Millipore, Ireland), aliquoted, and stored at 4°C until use. Since the fluorescence of dTomato fluorescent protein was lost after fixation, they were labeled with Alexa Fluor 594 NHS Ester (Thermo Fisher, USA, A20004) for visualization, according to the manufacturer's instructions. Alexa Fluor 594-labeled spores were then filtered through Steriflip Filter Units (Millipore, Ireland), aliquoted, and stored at 4°C until use.

### 2.3. Induction of Immunosuppression and Neutropenia

Immunosuppression was induced by treatment with a combination of cyclophosphamide monohydrate (Sigma-Aldrich, C0768) and cortisone 21-acetate (Sigma-Aldrich, C3103). Each mouse received 2 mg per mouse of cyclophosphamide at both four days prior and then one day prior to *A. fumigatus* conidium application. Cortisone acetate (2 mg per mouse) was administered one day prior to fungal spore application. Chemicals were dissolved in DPBS, followed by sonication at 37°C for 30 min. Intense shaking was used to enhance cortisone solubility.

Neutropenia was mimicked by injecting neutrophil-depleting antibodies, either anti-mouse Gr-1 (100 *μ*g per mouse; BioLegend, USA, clone RB6-8C5, 108414) or anti-mouse Ly6G (170 *μ*g per mouse; BioLegend, USA, clone 1A8, 127620). Antibody dosages were chosen according to published literature [8, 23]. Control groups received IgG2b (100 *μ*g per mouse; BioLegend, USA, clone RTK4530, 400622) or IgG2a (170 *μ*g per mouse; BioLegend, USA, clone RTK2758, 400516). All the antibodies and isotype controls were diluted in DPBS to a total volume of 200 *μ*l and administered via intraperitoneal injection 1 day prior to *A. fumigatus* conidium infection.

### 2.4. Application of *A. fumigatus* Conidia

Mice were anesthetized with l-chloro-2,2,2-trifluoroethyl difluoromethyl ether (Isoflurane, Abbott, UK). *A. fumigatus* conidia were dissolved in DPBS to a concentration of 1 × 10^8^ conidia/mL, and a 50 *μ*L aliquot containing 5 × 10^6^ conidia was applied to the oropharyngeal cavity of each mouse.

### 2.5. Tissue Processing and Whole-Mount Immunofluorescent Staining

Animals were euthanized and their lungs were inflation-fixed with 2% paraformaldehyde overnight. The main axial pathways of the left and right inferior lobes were microdissected. The left inferior lobe was used for specific immunostaining, while the right inferior lobe was used for staining with isotype antibodies. The airways were then washed with DPBS, permeabilized with 0.3% Triton X-100, and blocked with 1% bovine serum albumin (BSA; Serva, Germany) and 4% normal goat serum (Jackson Immuno Research, USA). Samples were immunostained as whole mounts with primary rat anti-mouse Gr-1 (1 : 50 dilution; BioLegend, USA, clone RB6- 8C5, 108402) or rat anti-mouse Ly6G (1 : 50 dilution; BioLegend, USA, clone 1A8, 127602). Following washing, Alexa 555-conjugated goat anti-rat IgG secondary antibody (1 : 250 dilution; Thermo Fisher, USA, A21434) was used for immunostaining. Where indicated, direct labeling with FITC-conjugated rat anti-mouse Gr-1 (1 : 50 dilution; Thermo Fisher, USA, clone RB6-8C5, 11-5931-81) or FITC-conjugated rat anti-mouse Ly6G (1 : 50 dilution; BioLegend, USA, clone 1A8, 127605) was performed after blocking with 2% BSA. All antibodies were diluted in 1% BSA. After the final washing step, samples were incubated with ActinRed (Thermo Fisher, USA, R37112) or SiRActin (Cytoskeleton Inc., USA, CY-SC001) for actin visualization and NucBlue (Thermo Fisher, USA, R37605) for nuclei visualization. All samples were mounted in Prolong Gold mounting medium (Thermo Fisher, USA, P36930).

### 2.6. Confocal Laser-Scanning Microscopy

An inverted confocal LSM780 microscope (Carl Zeiss, Germany) was used in all experiments with a 10x (NA = 0.3), 40x (NA = 1.4, water immersive), or 100x (NA = 1.46, oil immersive) objective, as indicated. Excitation at 405, 488, 561, and 633 nm was used to visualize Hoechst 33342, FITC/Alexa Fluor 488, Alexa Fluor 555/594, and SiRActin fluorescence, respectively. Emission was measured in CLSM *λ*-mode using a 34-channel QUASAR detector (Carl Zeiss, Germany) set to a 405–695 nm range. For quantitative analysis, images were captured as 2 × 2 tile grids at the same regions of each specimen using the 40x objective, with an individual xyz tile size of 354 *μ*m × 354 *μ*m × 20 *μ*m. Higher magnification images were acquired in *z*-stacks at the region of interest using the 100x objective. Spectral unmixing was performed using ZEN software by Zeiss. Finally, the images were processed using Adobe Photoshop (Adobe Systems, USA).

### 2.7. Quantitative Image Analysis

Image stacks were analyzed using Imaris software (Bitplane, Zurich, Switzerland). Neutrophils and *A. fumigatus* conidia along with the epithelial and smooth muscle layers were identified and processed via “three-dimensional surface rendering” of the appropriate channel, as previously described [9, 24]. The threshold and filter settings were optimized by visually comparing the result with the maximum-intensity projection. Based on the epithelium and smooth muscle layer position, the “Crop 3D” function was applied to each image to obtain the appropriate region for quantification. Neutrophil numbers were automatically calculated from the respective surface objects. Visual inspection was performed to confirm the accuracy of the automated quantitation results.

### 2.8. Statistical Analysis

Data are presented as scattered dot plots of the means or means ± SD for at least 3 mice. Differences between two groups were analyzed with the Mann–Whitney *U* test using GraphPad Prism version for Windows (GraphPad Software, USA, http://www.graphpad.com). A *p* value less than 0.05 was considered statistically significant.

## 3. Results

### 3.1. Identification of Neutrophils in the Conducting Airway Wall

**Figure 1:**
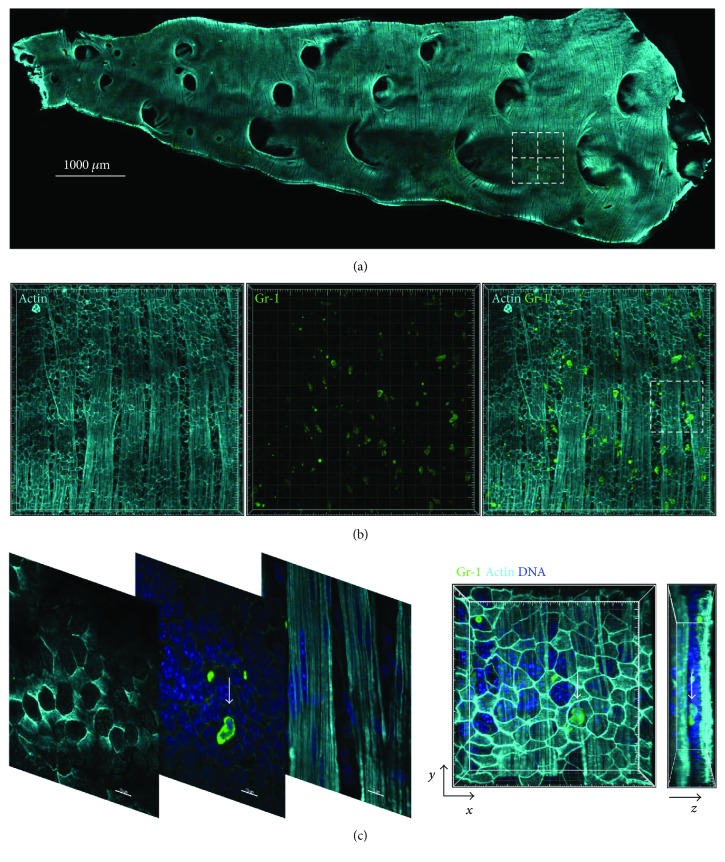
Identification of neutrophils in the airway wall. (a) Representative image of a microdissected whole-mount mouse conducting airway specimen stained for actin (SiRActin, light cyan) to visualize epithelial and smooth muscle cells and with anti-Ly6G/Gr-1 antibodies (green) to visualize neutrophils. Images from four segments (dotted line squares) were acquired as *z*-stacks for statistical analysis. Scale bar: 1000 *μ*m. (b) Higher magnification images of one of the segments indicated in (a) showing a fragment of the airway wall: epithelium and smooth muscles layer (left panel), neutrophils (green, middle panel), and merged image (right panel). Grid spacing: 20 *μ*m. (c) Higher magnification selective *z*-stack images of the region marked in (b) showing the epithelial layer (left image of *z*-stack), smooth muscle layer (right image of *z*-stack), and airway wall neutrophils (arrow, middle image of *z*-stack). The three-dimensional image shown in the far-right panel was generated by volume rendering of the *x*–*y* and *y*–*z* projections. An airway wall neutrophil is indicated with an arrow. Nuclei were stained with NucBlue (blue). Scale bar: 15 *μ*m; grid spacing: 5 *μ*m.

The airway wall, which starts from the luminal side of the epithelial border and is separated from the lung parenchyma by a layer of smooth muscle, undergoes structural remodeling as a result of chronic or short-term exposure to inflammatory stimuli [17]. Here, we investigated neutrophil localization in the conducting airway wall after a single inhalation of *A. fumigatus* conidia. To identify the airway wall compartment, we stained conducting airway whole-mounts for actin (Figures 1(a)–1(c), light cyan), as this protein is highly expressed by epithelial and smooth muscle cells. Notably, only the apical (or luminal) side of the airway epithelial cells was properly labeled with both ActinRed (a phalloidin-based dye) and SiRActin (a jasplakinolide-based dye). Neutrophils were identified based on Ly6G expression (Figures 1(b) and 1(c), green). Some neutrophils (Figure 1(c), arrows) were positioned between the luminal side of the epithelial border (Figure 1(c), left image of *z*-stack) and the smooth muscle layer (Figure 1(c), right image of *z*-stack). This observation was further highlighted in the lateral view of the three-dimensional volume rendering of the segment (Figure 1(c), *y*–*z* projection). Notably, these neutrophils were located in close proximity to other cells, whose position could be identified based on the location of their nuclei (Figure 1(c), middle image of *z*-stack).

### 3.2. Quantitative Analysis of Neutrophils in the Airway Wall of Infected Mice

**Figure 2:**
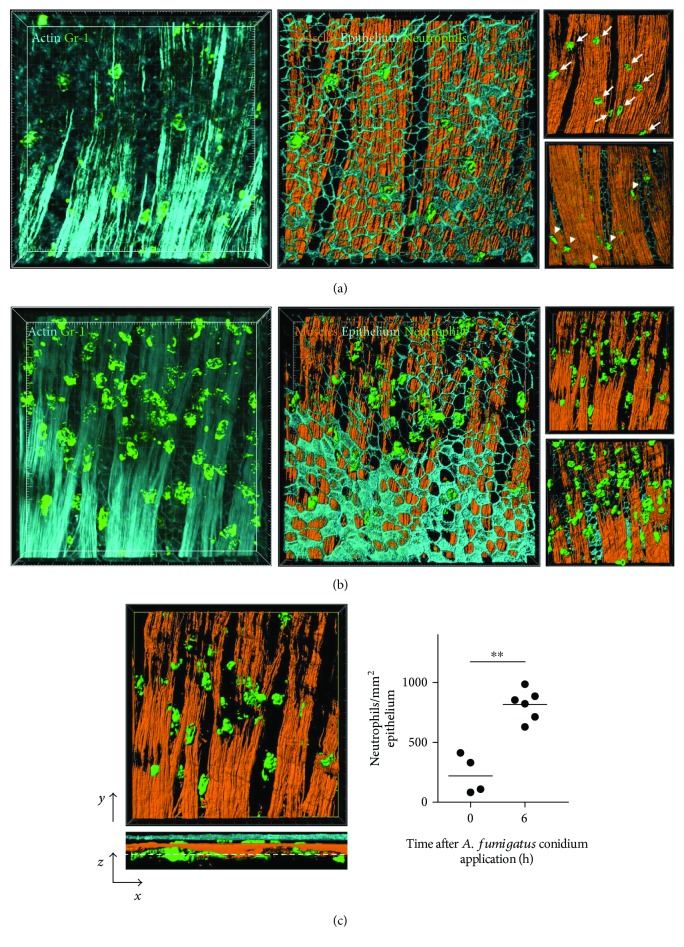
Distribution of neutrophils in the airway wall of intact and *A. fumigatus*-infected mice. (a–b) Representative maximum-intensity projection volume-rendering images (left panels) and surface-rendering images (middle and right panels) of the airway wall of intact mice (a) and *A. fumigatus*-infected mice 6 h after receiving conidia (b) showing the distribution of neutrophils (green) above the smooth muscle layer (ActinRed, light cyan or brown). Grid spacing: 20 *μ*m. In the surface rendering images, the epithelium (light cyan) was removed (upper-right panel) or the images were rotated 180 degrees (lower-right panel). Neutrophils located on the luminal side of the smooth muscle layer are highlighted by arrows (a) (upper right), while those below the smooth muscle layer are highlighted by arrowheads (a) (lower right). (c) Frontal (*x*–*y* projection) and side (*z*–*y* projection) views of the image represented in (b) (left) and quantitative analysis of the number of airway wall neutrophils in intact mice (0 h) and infected mice at 6 h after *A. fumigatus* conidium inhalation (right). The dotted line in the *z*–*y* projection indicates the arbitrary border of the airway wall. The data shown represent the independent experiments at 0 h (*n* = 4 mice) and at 6 h (*n* = 6 mice); each dot indicates the average of four measurements. ^∗∗^*p* < 0.01, Mann–Whitney *U* test.

Using three-dimensional images of the whole-mount conducting airway, we detected neutrophils in the airway walls of noninfected mice (Figure 2(a)) and mice infected with *A. fumigatus* conidia at 6 h after conidium application (Figure 2(b)). Epithelial and smooth muscle cells were labeled with actin, whereas neutrophils were labeled with Gr-1 (Figures 2(a) and 2(b), left panels). We generated three-dimensional objects, based on the three-dimensional images obtained via surface rendering. This approach allowed us to distinguish the actin-labeled epithelial and smooth muscle layers and to estimate the numbers of neutrophils located between and within these layers (Figures 2(a) and 2(b), middle panels). While not all neutrophils were found between the epithelial and smooth muscle layers (Figures 2(a) and 2(b), upper right), many were located in close proximity to the submucosal sides or between the smooth muscle layers (Figures 2(a) and 2(b), lower right). To exclude these submucosal neutrophils, analysis of each segment was performed using *x*–*z* projection (Figure 2(c), left panel). The abluminal surface of the smooth muscle layer served as the basis for *z*-cropping the segment. Quantitative analysis of the conducting airway wall neutrophils revealed a significant increase in airway wall neutrophil numbers at 6 h after *A. fumigatus* conidium challenge compared to that observed in noninfected mice (Figure 2(c), right).

### 3.3. Neutrophil–Conidium Interactions in the Conducting Airway Mucosa

**Figure 3:**
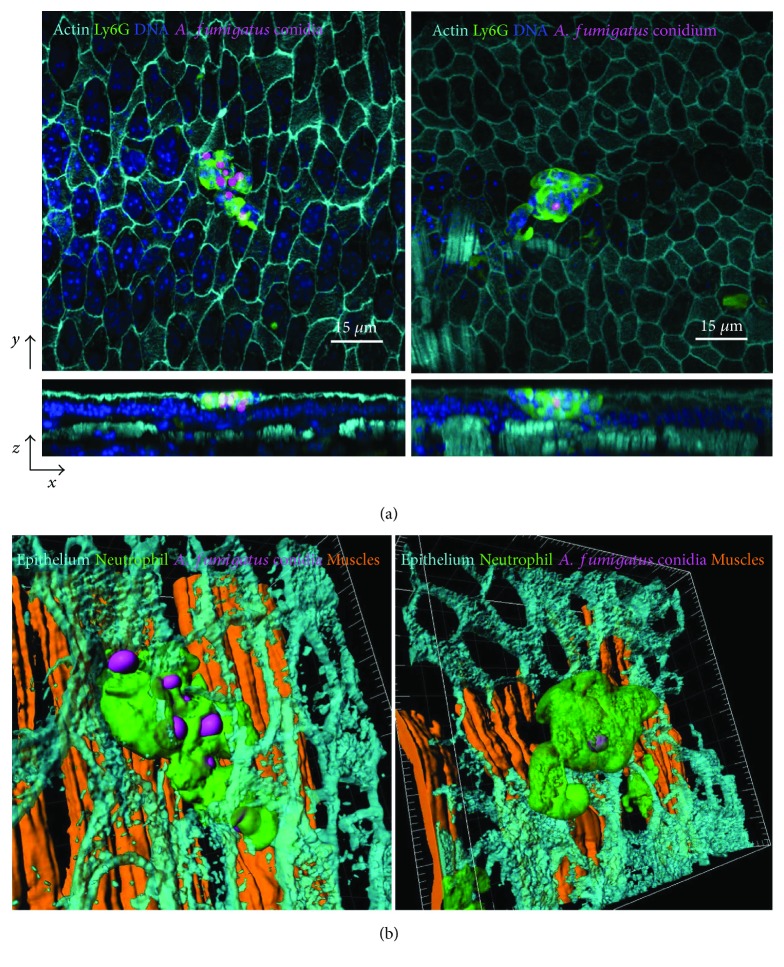
Internalization of *A. fumigatus* conidia by conducting airway wall neutrophils. (a) Frontal (*x*–*y* projection) and side (*z*–*y* projection) views of neutrophils (green) interaction with several (left panels) and single (right panel) conidia (magenta) in the conducting airway mucosa 6 h after the mice received *A. fumigatus* conidia; epithelium and smooth muscles are visualized by actin staining (SiRActin, light cyan) the nuclei (NucBlue, blue). Scale bar: 15 *μ*m. (b) Three-dimensional surface-rendering images of the enlarged-indicated areas showing neutrophils (green, transparent) that uptake conidia (magenta) in close proximity to the apical side of airway epithelium (light cyan); smooth muscles are indicated in brown. Grid spacing: 5 *μ*m.

Although small size of 3 *μ*m permits *A. fumigatus* conidia to penetrate small airways [25], upon oropharyngeal inhalation, conidia initially pass through the trachea and primary bronchi, where immune cells can sense and interact with them. Previously, we have shown that in case of acute allergic exacerbation, when neutrophil numbers in the airways were significantly increased as a result of allergen challenge, conducting airway neutrophils ingested conidia [9]. In the present study, we observed the cases of conducting airway neutrophil–conidium interactions after inhalation of conidia to intact mice. Massive recruitment of neutrophils was detected in order to localize both several conidia (Figure 3(a), left panel) and single spore (Figure 3(a), right panel). Precise analysis indicated that conidia always were bound by the neutrophils and the interaction occurred mainly in the luminal side of conducting airway epithelium (Figure 3(b)). Thus, uptake of conidia by neutrophils in conducting airway mucosa was observed in immunocompetent animals and such interaction can be necessary for providing resistance to *Aspergillus* infection in immunocompetent subjects.

### 3.4. Impact of Immunosuppression and Neutropenia on Neutrophil Number in the Airway Wall

**Figure 4:**
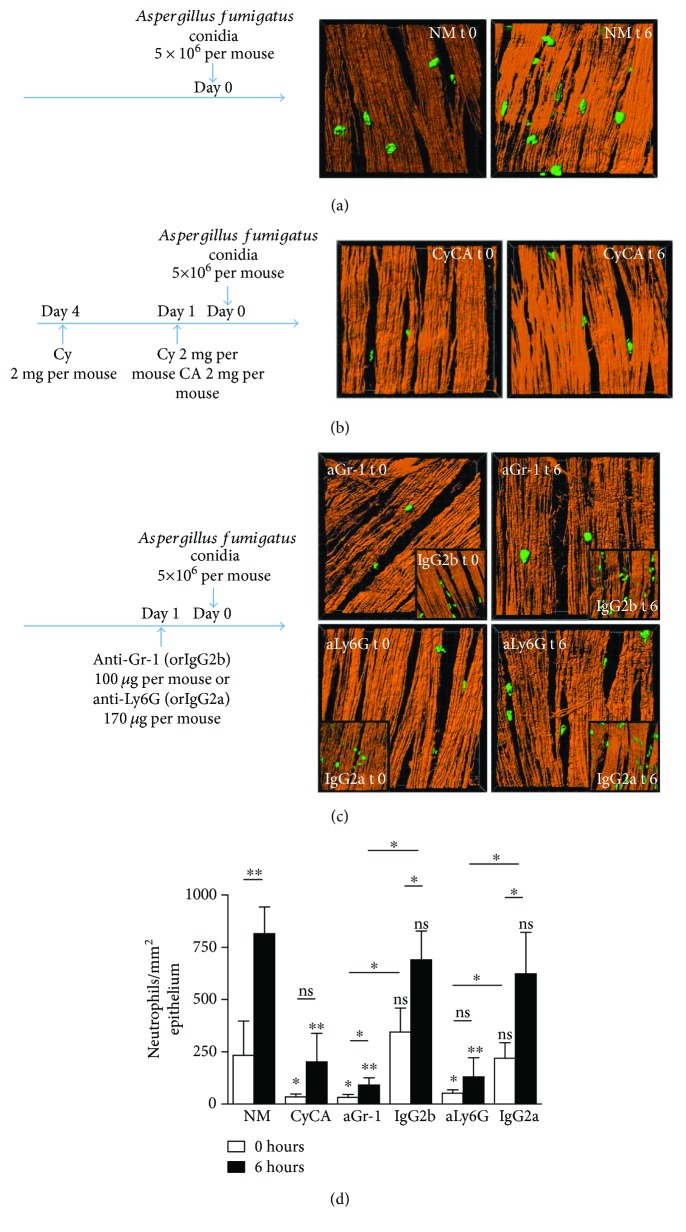
Effect of immunosuppression and neutrophil depletion on conducting airway wall neutrophils. (a–c) The experimental design (left) and representative surface rendering images of the conducting airway wall neutrophils (green) and smooth muscle layer (brown) before (left image panels) or 6 h after *A. fumigatus* conidium application (right image panels) in immunocompetent mice (NM) (a), mice induced by injections of cyclophosphamide (Cy) and cortisone acetate (CA) immunosuppression (CyCA) (b), and neutropenic mice that were treated with anti-Gr-1 (aGr-1) or anti-Ly6G (aLy6G) antibodies (c) along with mice that received the respective isotype controls (IgG2b or IgG2a). Quantitative analysis of the number of conducting airway wall neutrophils of each experimental group before conidium application (empty bars) and 6 h after (black bars) (d). The data are shown as the mean ± SD (*n* = at least 3 mice per group). The differences between the indicated groups were determined using the Mann–Whitney *U* test. ^∗^*p* < 0.05; ^∗∗^*p* < 0.01; ns: not significant.

Immunosuppression is a determinative aspect in a mouse model of invasive aspergillosis. In experimental mouse models, immunosuppression is commonly induced by injection of a combination of antineoplastic drugs and corticosteroids [26]. Alternatively, *in vivo* neutrophil depletion with anti-Gr-1 (clone RB6-8C5) or anti-Ly6G (clone 1A8) antibodies can also be performed to induce neutropenia [8]. In fact, depletion with anti-Gr-1 eliminates not only neutrophils but also minor populations of lymphocytes and dendritic cells, while anti-Ly6G antibodies target primary neutrophils and eosinophils [27]. Notably, neutrophil depletion appears to depend on the antibody concentration. Low doses of anti-Gr-1 as well as of anti-Ly6G do not deplete neutrophils from the circulation [28]. For anti-Gr-1, a single 80 *μ*g intraperitoneal injection to a 20 g mouse appears to induce peripheral blood neutropenia within 72 h [8]. The effects of Ly6G antibodies during inflammation are controversial in the field. For example, Wang et al. [29] demonstrated that Ly6G ligation inhibited the recruitment of neutrophils to the inflammation site (but did not affect the circulating neutrophil concentration), and Hasenberg et al. [30] reported that conditional Ly6G knockout did not affect neutrophil generation or migration to the site of inflammation. Susceptibility to single *A. fumigatus* conidium application was demonstrated both for mice with cyclophosphamide- and cortisone acetate-induced immunosuppression and for mice that received neutrophil-depleting antibodies. Thus, Svirshchevskaya et al. [31] showed the mortality and the presence of viable fungal spores in the lung homogenates at days 1, 3, and 5 after the application of 1 × 10^7^ conidia to mice treated with cyclophosphamide and cortisone acetate. Hartigan et al. [32] established fatal infection model by injecting mice with anti-Gr-1 24 hours prior to 6 × 10^6^* A. fumigatus* application. Later O'Dea et al. [33] observed lethal effect when *A. fumigatus* conidia were applied to mice with anti-Ly6G-induced neutropenia. Moreover, in both cases, the lung fungal burden was shown [32, 33]. The effects of immunosuppression or neutrophil depletion are commonly estimated based on peripheral blood leukocyte counts [23, 31]. In some cases, to confirm the absence of neutrophils after depletion at the site of inflammation, investigators have analyzed bronchoalveolar lavage fluids or lung homogenates [8, 32, 34]. Bruns et al. [7] showed that anti-Gr-1 treatment resulted in a decrease of interstitial neutrophils. Here, were determined the effects of immunosuppression and neutropenia on neutrophil counts in the conducting airway wall, both before and after *A. fumigatus* conidium application. Immunocompetent mice before and after infection served as controls (Figure 4(a)). Based on previous investigations [31, 34], we employed a common immunosuppression model that involves combination treatment with cyclophosphamide and cortisone acetate (Figure 4(b)). Previously established neutropenia models, induced by injection of either anti-Gr-1 [8, 32] or anti-Ly6G [8, 33], were also investigated (Figure 4(c)). The antibody dosages were chosen in accordance with previous literature [8, 23]. The control group received appropriate doses of IgG2b (an isotype control antibody for anti Gr-1) or IgG2a (an isotype control for anti-Ly6G) (Figure 4(c)). To ensure that anti-Ly6G application led to neutrophil depletion rather than competition of nonconjugated and FITC-conjugated anti-Ly6G by preventing the detection of recruited airway neutrophils, we used FITC-conjugated anti-Gr-1 for visualization; when depletion was performed with anti-Gr-1, reverse staining of the specimens was carried out with FITC-conjugated anti-Ly6G.

Immunosuppression as well as neutrophil depletion with either anti-Gr-1 or anti-Ly6G significantly reduced the number of conducting airway wall neutrophils in noninfected mice compared to their respective control-treated animals (Figure 4(d)). In the immunocompetent mice, oropharyngeal application of *A. fumigatus* conidia induced neutrophil recruitment to the conducting airway wall (Figures 4(a) and 4(c)), supporting our earlier data. Notably, 6 h after conidium application, the number of conducting airway wall neutrophils in mice with immunosuppression and both types of neutrophil depletion was significantly lower than that in immunocompetent animals (Figure 4(d)). Interestingly, a slight elevation in the number of neutrophils in the conducting airway wall was detected in mice with both immunosuppression and neutrophil depletion 6 h after conidium application compared to the respective noninfected control mice; however, this increase was not statistically significant (Figure 4(d)). Taken together, these data indicate that although some neutrophil recruitment to conducting airway occurs in immunosuppression and neutropenia, airway wall neutrophils are indeed susceptible to treatment with a combination of antineoplastic drugs and corticosteroids, as well as to antibody-mediated neutrophil depletion.

### 3.5. Gr-1^+^ and Ly6G^+^ Cells in the Conducting Airway Mucosa

**Figure 5:**
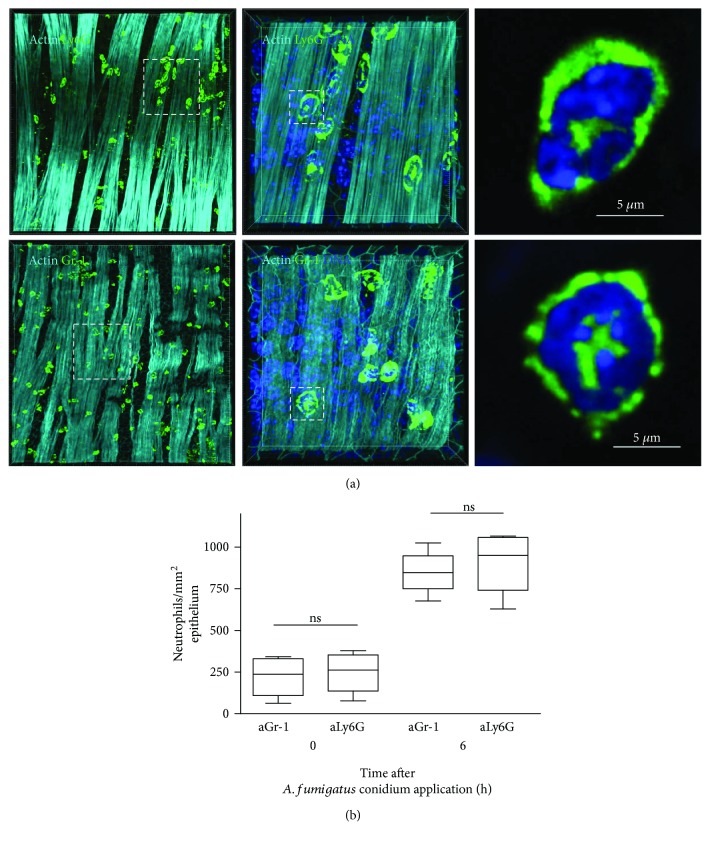
Identification of conducting airway wall neutrophils using anti-Ly6G or anti-Gr-1 antibodies. (a) Three-dimensional volume-rendering *z*-stack images of the conducting airway wall 6 h after the mice received *A. fumigatus* conidia at low (left panel, grid spacing: 20 *μ*m) and high (middle panel, grid spacing: 5 *μ*m) magnification showing Ly6G^+^ (upper row, green) or Gr-1^+^ cells (lower row, green), the nuclei (NucBlue, blue), and the smooth muscle layer (ActinRed, light cyan). Images of indicated neutrophils were magnified (right panels) and are presented as slices (scale bar: 5 *μ*m). (b) Quantitative analysis of Gr-1^+^ and Ly6G^+^ cells in the conducting airway of mice just before (0 h) or 6 h after *A. fumigatus* conidium application. The data shown represent the average of four measurements from each independent specimen (*n* = 4 mice). The data were analyzed using the Mann–Whitney *U* test. ns: not significant.

As reported above, we used anti-Gr-1 as detection antibodies to visualize conducting airway wall neutrophils in mice that received anti-Ly6G as depleting antibodies. Consequently, in the case of depletion with anti Gr-1, anti-Ly6G was applied for whole-mount airway immunostaining. Therefore, it was of great importance to compare the number of cells that were detected with anti-Gr-1 and with anti-Ly6G. Daley et al. [27] previously used flow cytometry to demonstrate that not all Gr-1-positive cells are neutrophils and that neutrophil identification with anti-Ly6G antibodies (clone 1A8) is more precise. However, Gr-1^+^ Ly6G^−^ cells are typically characterized by intermediate, rather than high, Gr-1 expression. With this knowledge, we sought to identify Gr-1^+^ and Ly6G^+^ cells in the conducting airway wall using LSM and compared the numbers of Gr-1^+^ and Ly6G^+^ cells present in both *A. fumigatus*-infected and noninfected airways. Our data demonstrate that both anti-Ly6G (clone 1A8) (Figure 5(a), upper row, green) and anti-Gr-1 (clone RB6-8C5) (Figure 5(a), lower row, green) antibody-stained cells possessed the typical neutrophil-surface-shaped contour (Figure 5(a), middle). Further analysis of these labeled cells identified a ring-shaped lobular nucleus that is specific for neutrophils [35] (Figure 5(a), upper- and lower-right panels). Visualization with anti-Ly6G (Figure 5(a), upper-middle and upper-right panels) as well as with anti-Gr-1 (Figure 5(a), lower-middle and lower-right panels) also revealed a common neutrophil spatial distribution of Ly6G [29].

To avoid the erroneous identification of anti-Gr-1-bound Ly6С^+^ cells as neutrophils, we quantified the numbers of anti-Gr-1- and anti-Ly6G-labeled cells in equal regions of the conducting airway wall of both noninfected mice and infected mice at 6 h after oropharyngeal application of *A. fumigatus* conidia. As shown in Figure 5(b), no significant difference was found between the number of neutrophils observed using anti-Gr-1 or anti-Ly6G antibodies. Thus, staining with either anti-Gr-1 or anti-Ly6G can be used for LSM identification of cells with a neutrophil-like morphology. Moreover, LSM shows only the cells that were previously found to express high levels of Gr-1 using flow cytometry [27].

## 4. Discussion

In the present study, using immunofluorescent staining of whole-mount airways and LSM, we showed the presence of a considerable amount of neutrophils in the conducting airway walls of intact mice. Previous findings demonstrated nondetectable/minor neutrophil levels in the bronchoalveolar lavages of intact mice, as well as minor Ly6G-positive cell populations in homogenized lungs [8, 9, 34, 36]. Baluk et al. [37] also identified few neutrophils in the tracheal mucosa of pathogen-free mice. In agreement with these studies, using immunofluorescent staining of whole-mount specimens, we observed neutrophils in the conducting airway mucosa and submucosa of intact mice. Moreover, LSM enabled us to quantify the neutrophils located in the airway wall—microcompartment between the epithelial and smooth muscle layers. The recent studies revealed the extended neutrophil lifespan [38] that allowed to suppose the presence of tissue-resident neutrophil pool. Alternatively, the neutrophils that we observed in the conducting airway wall in noninfected steady-state conditions were aged neutrophils that egressed from the bloodstream to the lungs [39]. However, the nature, the state, and the functionality of these neutrophils need to be further investigated.

Recruitment of neutrophils to the airway wall is associated with inflammatory conditions of varying origins [9, 11, 18, 19]. Using immunofluorescent staining of whole-mount airways and LSM, we investigated the recruitment and the localization of neutrophils in the conducting airways during *A. fumigatus* infection. Not surprisingly, fungal infection resulted in a significant increase in neutrophil recruitment in the conducting airway wall. We have also observed the events of conidium ingestion by neutrophils in the luminal side of conducting airway epithelium in immunocompetent mice. Together with reactive oxygen species production and neutrophil extracellular trap formation, phagocytosis is suggested to be a defense strategy of neutrophils against *Aspergillus* infection [40, 41]. Internalization of conidia by neutrophils was observed in the alveolar compartment of immunocompetent mice [7]. In accordance with this observation, our data support the essential role for conidium uptake by neutrophils at early stage of antifungal response. Such ingestion can contribute to maintain *Aspergillus* infection resistance that is observed in healthy peoples.

Since immunosuppression and neutrophil insufficiency-related disorders make patients more prone to fungal infections [4], in addition to establishing the migration of neutrophils to the conducting airway wall during *A. fumigatus* infection, we were also interested in determining whether conducting airway wall neutrophils are affected by immunosuppression or depleting antibody-induced neutropenia. It is important to note that to reach the airway wall, neutrophils from the blood stream must cross the endothelial barrier, endothelial basement membrane, smooth muscle layer, and epithelial basement membrane in order to fit between the epithelial cells. This migration path leads to neutrophil maturation, which involves changes in morphology and surface marker expression [42, 43]. In addition, the airway wall could provide a safe anatomical niche preventing the retrieval of airway wall neutrophils in contrast to circulating neutrophils that are affected by cortisone acetate and cyclophosphamide [31]. In the case of neutrophil-depleting antibody treatment, we also expected that neutrophils between the epithelial and smooth muscle layers could be protected from efferocytosis following Ly6G- or Fc*γ* receptor linking [30, 44]. Furthermore, Moses et al. [45] observed residual neutrophil survival after depletion with anti-Ly6G. Thus, it is also possible that the residual neutrophils observed in the studies described above were safely harbored in the airway wall during immunosuppression and neutrophil depletion. In the present study, we detected neutrophils in the conducting airway wall of mice that were treated with a combination of cyclophosphamide and cortisone acetate or neutrophil-depleting antibodies (anti-Gr-1 or anti-Ly6G). Neutrophil surface renderings were generated based on three-dimensional confocal images, allowing precise quantitation of airway wall neutrophils. These data indicate that the amount of conducting airway wall neutrophils in mice following immunosuppression or neutropenia was significantly lower compared to that in immunocompetent mice. Interestingly, in response to a single application of 5 × 10^6^* A. fumigatus* conidia, we observed de novo neutrophil recruitment to the conducting airway wall in all infected mice. While this increase in the immunocompetent mice 6 h after conidium application was significant compared to time 0, in mice with immunosuppression or neutropenia, the trend was strong, but not statistically significant. It should be noted, however, that this increase in neutrophil numbers in the conducting airway 6 h after conidium application in the immunosuppressed and neutropenic mice was still significantly lower than that in immunocompetent infected mice.

It has been reported [8] that both immunosuppression and neutrophil depletion can promote the retrieval of circulating neutrophils for up to 72 h after treatment. As the present study was focused on changes much earlier than this threshold, it is unlikely that the neutrophils recruited to the conducting airway wall in the immunosuppression and neutropenia models were circulating neutrophils, but were presumably from a resistant marginated vascular neutrophil pool. Taken together, our data indicate that conducting airway wall neutrophils appear to be sensitive to both immunosuppression and antibody-mediated depletion and that inhalation of fungal spores leads to the de novo recruitment of neutrophils to this microcompartment from a marginated vascular pool.

## 5. Conclusions

Our results demonstrate the presence of neutrophils in the conducting airway wall in the noninfected steady state. Notably, these neutrophils were not resistant to immunosuppression or antibody-mediated neutrophil depletion. Upon *A. fumigatus* infection in immunocompetent mice, the population of airway wall neutrophils significantly increased, likely due to circulating neutrophils infiltrating into the tissue. An elevation in the number of conducting airway neutrophils was also observed in immunosuppressed and neutropenic mice in response to fungal infection. Although the alteration was not statistically significant, it suggests the presence of immunosuppression- and neutrophil depletion-resistant neutrophil populations in compartments other than the airway wall. While additional work is necessary to further investigate this phenomenon, this study provides valuable insight into the infiltration of neutrophils in the conducting airway wall, which enhances the understanding of immune responses to *A. fumigatus* infection.
